# Immigration ensures population survival in the Siberian flying squirrel

**DOI:** 10.1002/ece3.2807

**Published:** 2017-02-15

**Authors:** Jon E. Brommer, Ralf Wistbacka, Vesa Selonen

**Affiliations:** ^1^ Department of BiologyUniversity of TurkuTurkuFinland; ^2^ Department of BiologyUniversity of OuluOuluFinland

**Keywords:** dispersal, gliding mammal, integrated population model, mark–recapture, population growth

## Abstract

Linking dispersal to population growth remains a challenging task and is a major knowledge gap, for example, for conservation management. We studied relative roles of different demographic rates behind population growth in Siberian flying squirrels in two nest‐box breeding populations in western Finland. Adults and offspring were captured and individually identifiable. We constructed an integrated population model, which estimated all relevant annual demographic rates (birth, local [apparent] survival, and immigration) as well as population growth rates. One population (studied 2002–2014) fluctuated around a steady‐state equilibrium, whereas the other (studied 1995–2014) showed a numerical decline. Immigration was the demographic rate which showed clear correlations to annual population growth rates in both populations. Population growth rate was density dependent in both populations. None of the demographic rates nor the population growth rate correlated across the two study populations, despite their proximity suggesting that factors regulating the dynamics are determined locally. We conclude that flying squirrels may persist in a network of uncoupled subpopulations, where movement between subpopulations is of critical importance. Our study supports the view that dispersal has the key role in population survival of a small forest rodent.

## 1 Introduction

The population growth rate is determined by survival, juvenile production, and dispersal patterns of the species. Immigration and emigration (individuals moving in and out of the population, respectively) have the potential to play a central role in the population ecology of species (Clobert, Baguette, Benton, & Bullock, 2012; Thomas & Kunin, 1999). However, immigration and emigration are also typically the most difficult demographic rates to estimate. Our understanding of dispersal, the process behind immigration and emigration, has increased tremendously during recent decades (Clobert et al., 2012; Nathan et al., 2008; Ronce, 2007). At large spatial scales, dispersal can synchronize the dynamics of separated populations (Liebhold et al., 2004; Ranta, Kaitala, Lindström, & Linden, 1995). Still, linking movement to population dynamics (Bowler & Benton, 2005; Morales et al., 2010) has remained a challenging task and conservation management suffers from poor knowledge on dispersal (Driscoll et al., 2014). Indeed, attempts to link dispersal and population dynamics have been limited to certain taxa, in vertebrates to birds and large ungulates (e.g., Coulson, Albon, Pilkington, & Clutton‐Brock, 1999; Grøtan et al., 2009; Lampila, Orell, Belda, & Koivula, 2006; Schaub, Jakober, & Stauber, 2013).

There are currently three main approaches used to link movement to population dynamics. The first approach uses models that are linked to the spatial and temporal dynamics of populations (Morales et al., 2010; Revilla & Wiegand, 2008; Risk, de Valpine, & Beissinger, 2011). This approach is often based on detailed data on movement, for example, through GPS tracking, and individual‐based modeling. A second approach is to rely on genetic approaches, which allow making inferences on connectivity between populations also in systems where direct measures of movement are highly challenging to obtain (Hamrick & Trapnell, 2011; Lowe & Allendorf, 2010). A third approach relies on recent advances in mark–recapture analysis (Royle, Chandler, Sollman, & Gardner, 2013). An exciting development of this approach is the integration of information from multiple sources into a unified hierarchical modeling framework. This development is recognized as a fruitful approach for gaining an understanding of population ecology (e.g., Brooks, Freeman, Greenwood, King, & Mazzetta, 2008; Buckland, Newman, Thomas, & Koesters, 2004; King, Brooks, Mazzetta, Freeman, & Morgan, 2008; Schaub, Gimenez, Sierro, & Arlettaz, 2007).

One group of mammalian species with conservation interests all around the world is gliding mammals, which include flying squirrels and gliders (Lindenmayer et al., 2011; Selonen, Sulkava, Sulkava, Sulkava, & Korpimäki, 2010; Smith, Gende, & Nichols, 2005). Substantial fluctuations in population size, including cyclical dynamics and declines, have been observed in the dynamics of flying squirrel species (Fryxell, Falls, Falls, & Brooks, 1998; Lampila, Wistbacka, Mäkelä, & Orell, 2009). One reason for declining trends in abundance of many gliding mammals is that this species group is often specialized to forest habitats that include cavities for nesting. In addition, gliding mammals typically are on the “slow” end of the life‐history spectrum, including traits such as low mortality, low metabolic rate, and few offspring per litter (Fokidis & Risch, 2008; Lindenmayer et al., 2011; Smith, 2007). On the other hand, some gliding mammals may have longer dispersal distances than other similar‐sized mammals (Hanski & Selonen, 2009). Individuals may hence be able to live and move in relatively fragmented landscapes (Selonen & Hanski, 2010, 2012). Nevertheless, declining trends (Selonen et al., 2010; Smith et al., 2005) and sudden local extinctions (Lindenmayer et al., 2011) have been observed.

**Figure 1:**
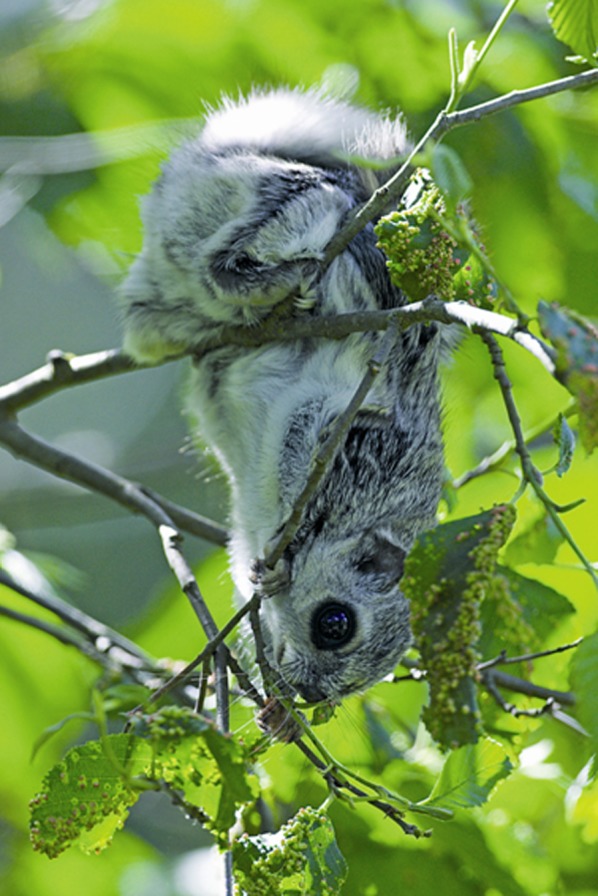
The study organism, Siberian flying squirrel *Pteromys volans*. Photograph by Henrik Lund

In this study, we use long‐term mark–recapture data for two separate nest‐box breeding populations of Siberian flying squirrels, *Pteromys volans* (Figure 1), which were individually identifiable. We aim to (i) compare the importance for population growth of immigration relative to rates of survival and fecundity. To this end, we construct an integrated population model (IPM) for two separate populations of ear‐tagged Siberian flying squirrels, combining information on (1) population census (number of recorded nests with offspring), (2) fecundity, and (3) apparent survival of juvenile and adult females derived from capture–recapture analysis. Comparison of which demographic rates correlate with population growth rates in two populations is a particularly powerful design for identifying factors regulating population dynamics. This is because any regulatory factors found to be operational in one population may be specific to this one study population only. Thus, we expect that demographic rates which are key to flying squirrel population dynamics are prominent correlates of population growth rate in both our study populations. We further explore whether predominantly local processes or larger‐scale processes regulate flying squirrel dynamics. Larger‐scale processes such as environmental forcing (Moran, 1953; Royama, 1992) , or trophic interactions (e.g., Korpimäki, Norrdahl, Huitu, & Klemola, 2005) can drive synchrony in population dynamics between separate populations. We therefore (ii) test whether demographic rates are primarily dependent on local population size (i.e., density dependent), and (iii) whether demographic rates and population dynamics in our two spatially separated populations are correlated.

## 2 Material and Methods

### 2.1 Study species

The Siberian flying squirrel (Figure 1) is an arboreal rodent, which nests in tree cavities, nest boxes and dreys in spruce‐dominated boreal forests. Female flying squirrels are mostly territorial, living in separate on average 7 ha home ranges, but males live in overlapping on average 60 ha home ranges encompassing several males and females (Selonen, Painter, Rantala, & Hanski, 2013). Population redistribution occurs during the offspring natal dispersal period in autumn, as breeding dispersal is rare in flying squirrels (Hanski & Selonen, 2009). Distances moved during natal dispersal are typically 1–2 km and are not much affected by landscape structure (Selonen & Hanski, 2004, 2012). The Siberian flying squirrel is strictly protected under European legislation (Habitats Directive 92/43/EEC).

### 2.2 Study population and methods

Flying squirrels were studied by setting up nest boxes in two study areas. One study area (Luoto) is situated on a peninsula in western Finland (63°49′N, 22°49′E). The other study area (Vaasa; 63°3′N, 22°41′E) is situated approximately 100 km south of Luoto. In Luoto, flying squirrels were studied within an area of 44 km^2^. The main forest types in Luoto are shoreline spruce‐dominated mixed forests, clear‐cuts, and cultivated Scots pine forests. The Vaasa study area was 25 km^2^ and is covered by spruce forest patches, clear‐cuts, and agricultural fields. Nest boxes were placed in forest patches of various sizes (1–25 ha) in sets of 2–4 nest boxes per site, on average two nest boxes per forest hectare. More information on the study areas is provided by Lampila et al. (2009) and Selonen, Hanski, and Wistbacka (2014). Most forests in Finland are commercially managed, and natural cavities are not abundant. Indeed, most forest patches in our study area totally lack natural cavities and cavity density is on average 0.1 cavities per hectare based on 742 spruce forest hectares surveyed within our study areas. The first nest boxes in the study areas were established in 1989. Collection of the material analyzed in this paper started in 1992 in Vaasa and 1993 in Luoto. However, the box network continued to grow and methodology was refined which hinders comparison between the populations and the evaluation of unbiased time trends. In this study, we use information from the Vaasa population starting in 2002 and from the Luoto population starting in 1995. Information up to 2014 was included. We only considered nest‐box plots which were surveyed for the whole study period, and thus were comparable in terms of monitoring effort. This was done to assure that the time series of population census is representative of the dynamics of the number of flying squirrels occurring in the nest boxes. There were approximately 250 nest boxes in both Vaasa and Luoto during the study period, which were included in this study. We refer from here onwards to these two nest‐box networks as study populations. There was thus a temporally variable number of nest boxes in Vaasa and Luoto, which were controlled (i.e., checked and animals were tagged), but which we here did not consider part of the study population and were hence ignored. Given the relatively modest number of flying squirrels living in these boxes (see “3”), we do not consider the flying squirrel population to be restricted by nest‐box availability.

Nest boxes were checked in June each year. Occupied sites were again checked in August because flying squirrel females can have two broods per year. Flying squirrels use boxes for roosting and may use more than one box for raising broods. Thus, we might underestimate the number of second litters, if females changed nest after the first brood. This sometimes happens as females may avoid parasites by nest change. However, within female territories there were more than one nest box present, and we are unaware of observations that females would give a birth in a drey, as new born litters are only observed from natural cavities or nest boxes (own observations). This increases our likelihood to locate the summer litters. Box occupancy percentage was low (~25%); that is, in most cases a nest box was empty when checked, indicating that available boxes were not limiting our study population. When encountered, juvenile and adult flying squirrels were captured from nest boxes, sexed, weighed, and tagged in order to allow individual identification by placing a unique tag in the ear of the individual. A few offspring were not sexed because they were too small. Female flying squirrels occurring together with juveniles in the same nest box were assumed to be the juveniles’ mother. Some tags were likely lost (based on the absence of a tag and a damaged ear). Tags were replaced when needed, and—whenever possible—the identity of the individual with tag loss was inferred on the basis of territory occupancy or other characteristic. For a subset of the collected material, we could estimate that the individual identity was lost due to tag loss in approximately 3% (8/266) of interannual recaptures (calculated as the sum of adults captured with a broken ear and of tagged adults). Formal inclusion of the probability of tag loss can be included in mark–recapture models, but doing so requires a design where individuals are marked with at least two separate marking methods (Pollock, Nichols, Brownie, & Hines, 1990). We ignored tag loss in the models described below, and our estimate of survival was hence slightly downward‐biased.

### 2.3 Integrated population model

An IPM is a hierarchical model for population‐level analysis of individual‐based data (e.g., Besbeas, Freeman, Morgan, & Catchpole, 2002). An IPM may infer demographic rates, which have not been observed directly, including immigration rate (Schaub & Abadi, 2011). We constructed an IPM closely following the models outlined by Abadi, Gimenez, Ullrich, Arlettaz, and Schaub (2010), Kéry and Schaub (2012), and Schaub and Fletcher (2015). In general, the IPM followed standard reasoning of hierarchical models in population ecology (Kéry & Schaub, 2012; Royle & Dorazio, 2008). Hierarchical models assume that latent (unknown) state variables (in this case, population sizes of 1‐year‐old, adult and immigrant individuals in the population) undergo a specific state process (in this case, population dynamics). Latent states are then linked to observations assuming a specific observation process, which allows deriving information on the latent states themselves, including stages such as immigrants which are not directly observed.

Details of the IPM are provided in the electronic supplement. Briefly, population size was defined as the number of females. Changes in population size in the study populations arose because of four demographic processes: survival (an individual alive in the study population at time *t* survives to be present in time *t *+ 1), fecundity (number of offspring leaving the nest at time *t*), immigration (an individual born outside the study population establishes herself in the study population at time *t*), and emigration (an individual moves outside the study population between *t* and *t *+ 1). We distinguished the number of locally recruited 1‐year‐old female flying squirrels from the number of females older than 1 year (adults) that remained in the study population, and (1 year old or older) females which immigrated into the study population. Estimates of annual apparent survival was based on capture–recapture analysis of ear‐tagged individuals, separately estimating first‐year apparent survival (based on tagged young) and adult apparent survival (based on all tagged females). In general, capture–recapture analysis uses the history of whether marked individuals are encountered in each year of the study to separate the probability of capture from the probability of apparent survival (Lebreton, Burnham, Clobert, & Anderson, 1992). Apparent survival estimates the combined effects of true survival and emigration out of the study population, and this metric hence implicitly includes the probability to leave the study population (emigration). Annual population census was based on the number of nests with offspring identified, irrespective of whether offspring could be ear‐tagged or the female was identified. Because a flying squirrel female can have two broods in one season, the number of nests with offspring can both underestimate and overestimate the true number of females present in the study population. Annual reproduction was estimated based on the number of female offspring ear‐tagged (and which hence survived to relative old age) produced in all nests where also individual females were identified. A few offspring were not sexed and were counted as 0.5 female each, assuming equal sex ratios. The annual number of immigrants was estimated on the basis of the difference between the latent population size (derived largely from the population census) and what the latent population size would be in case fecundity and apparent survival of juveniles and adults were the only demographic processes (cf. Abadi et al., 2010). Thus, estimates of the annual number of immigrants were completely model based and did not require any assumptions or direct information regarding immigrants from data. Immigration rate at time step *t* was calculated as a derived parameter by dividing the number of immigrants arriving in the study population at time *t *+* *1 by the population size at time *t* (Schaub & Fletcher, 2015).

The model was implemented in a Bayesian framework in WinBUGS (Lunn, Thomas, Best, & Spiegelhalter, 2000) by adapting code detailed by Kéry and Schaub (2012). Population sizes in the first time step of 1‐year‐old, adult and immigrant females were randomly drawn from a normal distribution with a mean of 10 and standard deviation of 100, truncated to be positive. We used vague priors for the logit of mean juvenile and adult apparent survival and capture rates and the log of mean fecundity, and uniform priors for the standard deviations associated with the random effects that modeled between‐year differences in these parameters. The number of animals immigrating into the population was estimated for each year separately and was given a uniform prior in the interval of −10 to 30 (Schaub & Fletcher, 2015). In practice, WinBUGS was called from R implemented using the package R2WinBUGS (Sturtz, Ligges, & Gelman, 2005), and the script for the WinBUGS model used is presented in Appendix S2.

Model posteriors were summarized using their posterior mode as a description of the expected value, and using their highest posterior density as the 95% credible interval (CRI), which were both derived from a density kernel (Hadfield, 2010). This description of the posteriors was used as many of the model parameters (e.g., variances) were constrained to be positive values and hence posterior distributions were often skewed.

### 2.4 Analysis of model‐derived estimates of demography and population growth

The above‐outlined IPM produced estimates of annual population growth rates. In addition, we calculated the geometric mean population growth rate over the entire study period. As the IPM was implemented in a Bayesian framework, the uncertainty in these measures of population growth rate is taken forward using the entire posterior distribution. The importance of each of the demographic rates for annual population growth rate was examined by calculating the posterior density of the correlation between each of the demographic rates and population growth rate. The proportion of all correlations between the posteriors which was larger than zero was calculated. We considered correlations between a demographic rate and annual population growth rate that have a probability greater or equal to .975 of either being positive or negative as evidence of a significant relationship.

### 2.5 Density dependence

We investigated density dependence by correlating the posteriors of total population size at time *t* with the demographic and population growth rates during time step *t* to *t *+* *1. We used this approach (cf., Schaub et al., 2013) instead of modeling density dependence as part of the IPM, because there are no data on immigration in the IPM, and hence, density dependence in this inferred property cannot be estimated as part of the IPM. Because posteriors of total population size are derived from a model correcting for detection probability, this procedure avoids spurious documentation of density dependence, which one would risk when using uncorrected counts (Freckleton, Watkinson, Green, & Sutherland, 2006; Knape & de Valpine, 2012). Because immigration rate is a function of the reciprocal of total population size, we also calculated the relationship between the number of immigrants entering the population at time *t *+* *1 and total population size at time *t*.

## 3 Results

### 3.1 Population trends

**Figure 2:**
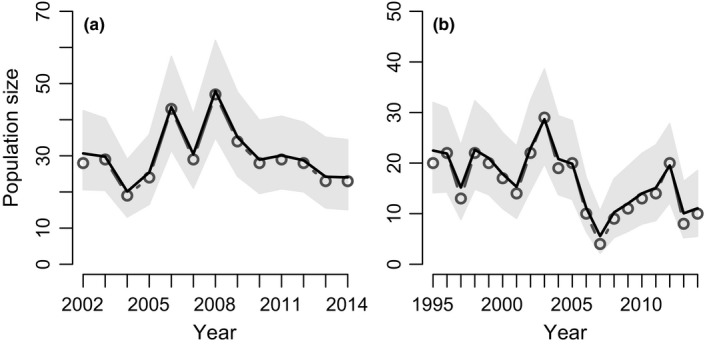
Integrated population model (IPM)‐derived estimates of population size in the study populations (a) Vaasa and (b) Luoto. Plots show the census (line with dots; “*y*” in Table 1) and IPM‐estimated population size (black with its 95% CRI in gray)

A total of 1,163 and 993 female juveniles and adults were ear‐tagged in the study populations in Vaasa and Luoto, respectively. The census population sizes of both study populations, as well as the number of females observed to reproduce and the number of daughters they produced, clearly varied during the course of the study (Table 1). The IPM‐derived estimates of population sizes and their 95% CRI revealed that in the Vaasa study population (Figure. 2a), population sizes fluctuated around 31 ≥1‐year‐old females throughout the study period. Geometric mean population growth rate over 13 years indicated fluctuations around a stable population size (Table 2). In the Luoto study population (Figure 2b), the population size reached a peak in 2003 (estimated at 28.7, 95% CRI [20.2, 38.9] females), but declined drastically in only 4 years (to 5.6 females in 2007, 95% CRI [2.1, 10.5]). Over the study period, the population size in the Luoto study population declined slightly (negative geometric mean population growth rate, Table 2). When the equivalent period (2001–2014) was considered, the decline in the Luoto was not significant; the geometric mean population growth rate (pgr) during this period was 0.97, 95% CRI [0.92, 1.04].

**Table 1:** Census of the population (*y*, number of broods found), and the total number of tagged daughters (*J*) produced by the number of reproductive females identified (*R*), for each year of the study for the two study populations. See Appendix S1 for details of how these parameters were used in the IPM

Year	Vaasa			Luoto		
	*y*	*J*	*R*	*y*	*J*	*R*
1995				20	21	19
1996				22	19	18
1997				13	12	12
1998				22	22	17
1999				20	22	19
2000				17	17.5	13
2001				14	19	13
2002	28	27	21	22	20	19
2003	29	25	20	29	30	24
2004	19	8	13	19	16	17
2005	24	23	18	20	20	15
2006	43	45	35	10	12.5	9
2007	29	27	23	4	3	2
2008	47	33.5	32	9	4	4
2009	34	27	26	11	8	10
2010	28	19	18	13	12.5	12
2011	29	17	20	14	14.5	11
2012	28	24	16	20	25.5	16
2013	23	11	11	8	6	8
2014	23	17	15	10	12	10

**Table 2:** Estimates of mean and standard deviation σ, denoting the between‐year fluctuations around the mean, for all demographic parameters of the integrated population model (IPM). The 95% credible interval (CRI) of all parameters is provided within square brackets. The mean and CRI of the geometric mean population growth rate (GM pgr) is provided as a derived parameter from the IPM. The immigration rate is plotted in Figure 2

	Vaasa		Luoto	
	Mean [95% CRI]	σ [95% CRI]	Mean [95% CRI]	σ [95% CRI]
Juv.surv	0.13 [0.086, 0.21]	.43 [0.03, 1.31]	0.16 [0.12, 0.22]	.038 [0.0011, 0.69]
Ad.surv	0.36 [0.26, 0.42]	.18 [0.000074, 0.78]	0.47 [0.41, 0.54]	.27 [0.0016, 0.72]
Fecundity	1.13 [0.98, 1.28]	.029 [0.00025, 0.24]	1.13 [1.00, 1.28]	.022 [0.00052, 0.17]
Capture prob.	0.85 [0.71, 0.99]	.99 [0.0016, 2.7]	0.77 [0.65, 0.87]	.14 [0.0030, 1.26]
GM pgr	0.98 [0.93, 1.03]		0.97 [0.93, 0.99]	

### 3.2 Demographic rates

**Figure 3:**
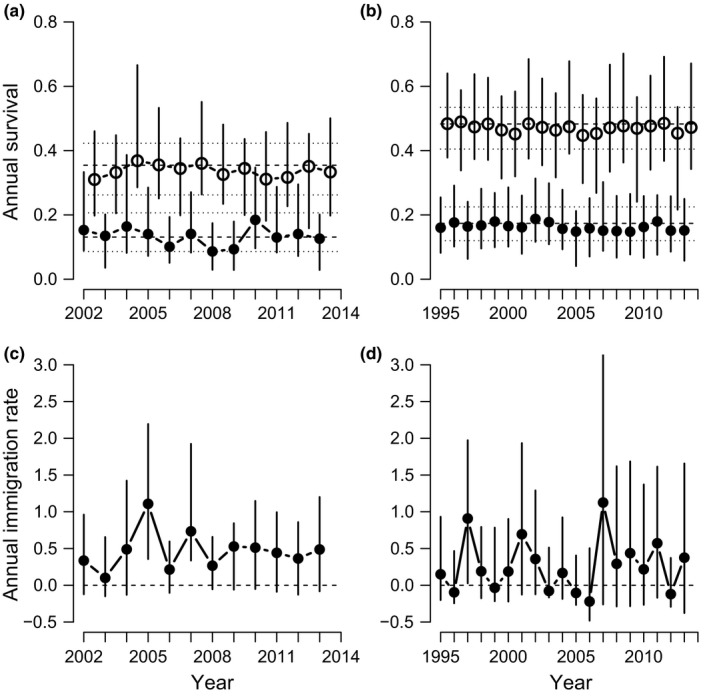
Apparent annual survival and immigration probabilities for the population Vaasa (a, c) and Luoto (b, d). Panels (a, b) show the posterior modes of apparent survival for juveniles (filled dots) and adults (open dots) with bars indicating their 95% CRI. Overall average apparent survival is indicated by a dashed line and its 95% CRI by dotted lines. (c, d) Annual immigration rate (number of females immigrating into the population in the following year per ≥1‐year‐old females present this year) with its 95% CRI

The mean demographic rates in both study populations were qualitatively the same, in the sense that their 95% CRIs overlapped (Table 2, Figure 3). Adult survival tended to be lower and immigration rate tended to be higher in the study population in Vaasa compared with the study population in Luoto. Mean adult survival probability was about 35%–50% in both study populations and mean juvenile survival was approximately 15%–20% (Table 2; Figure 3a,b). The uncertainty around the annual estimates of immigration rate was high (Figure 3c,d) because it was fully inferred by the IPM. Fecundity was largely constant across years in both populations (Table 2, Figure S1).

### 3.3 Consequences for population growth rate

**Figure 4:**
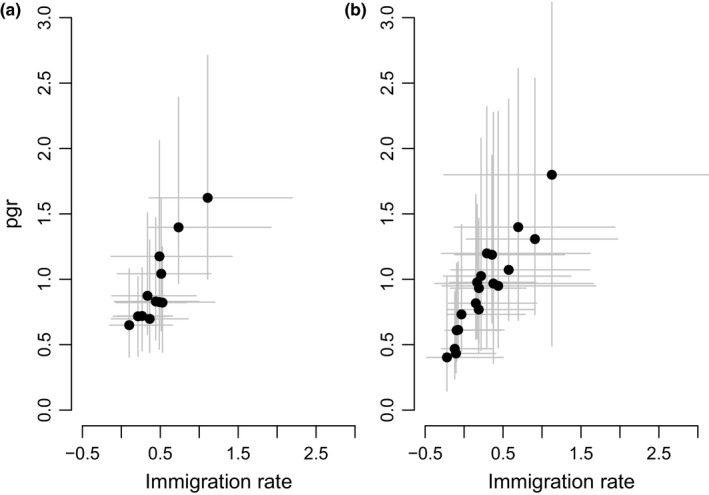
Relationship between model‐derived annual population growth rate (pgr) and immigration rate for all the study years in the Vaasa (a) and Luoto (b) populations. Each plot shows, as filled dots, the annual posterior mode of population growth rate plotted against the annual posterior mode of immigration rate. The 95% CRI of annual population growth rate is shown as a vertical bar, whereas the 95% CRI of immigration rate is shown as a horizontal bar. See Table 3 for statistics on the correlation coefficients based on these model output. Similar plots showing the correlation of all demographic rates to population growth rate are presented in Figures S2 and S3

Most demographic parameters (survival of juveniles and adults, fecundity, and immigration rate) correlated positively with pgr (Table 3a, Figures S2, S3). In both study populations, immigration rate showed a strong correlation with population growth rate (Figure 4a,b). Adult, but also juvenile survival in the study population in Vaasa correlated reasonably high with pgr, but the associations between juvenile and adult survival and pgr were markedly less strong in the study population in Luoto (Table 3a), indicating that the association of these vital rates with pgr was not a robust finding across populations.

**Table 3:** The correlation and its 95% CRI between annual values for different parameters (par) underlying the population dynamics and (a) the annual population growth rate and (b) population size. The correlations with population size test for density dependence and note that here the number (Nr) of immigrants was used as well as population growth rate (pgr). Correlations with a probability >95% of being positive or negative are indicated in bold, and these we consider providing strong evidence for either being a driver of pgr (in a) or for undergoing density dependence (in b). The correlation between immigration rate and pgr is plotted in Figure 3. All correlations between demographic rates and pgr are plotted in Figures S2 and S3 for the Vaasa and Luoto population, respectively. The correlations between population sizes and immigration rates and pgr are plotted in Figure S4

Parameters	Vaasa				Luoto			
	Correlation	Lower 95% CRI	Upper 95% CRI	*p* (*r* > 0)	Correlation	Lower 95% CRI	Upper 95% CRI	*p* (*r* > 0)
(a) Correlation with population growth rate								
Juvenile survival	.33	−0.21	0.78	.84	.046	−0.43	0.47	.58
Adult survival	.35	−0.35	0.75	.79	.16	−0.30	0.61	.77
Fecundity	.079	−0.58	0.57	.48	.10	−0.44	0.44	.49
Immigration rate	**.96**	**0.80**	**0.99**	**.99**	**.95**	**0.84**	**0.99**	**1**
(b) Correlation with population size								
Juvenile survival	–.45	–0.75	0.20	.11	.11	–0.37	0.52	.65
Adult survival	–.22	–0.66	0.42	.38	–.029	–0.55	0.37	.41
Fecundity	.13	–0.49	0.63	.66	.061	–0.43	0.50	.53
Nr of immigrants	–.43	–0.65	0.20	.15	–.19	–0.52	0.068	.085
Immigration rate	–**.49**	–**0.78**	–**0.25**	**.002**	–**.56**	–**0.74**	–**0.31**	<**.001**
pgr	–**.63**	–**0.77**	–**0.41**	**0**	–**.55**	–**0.69**	–**0.39**	**0**

### 3.4 Density dependence

We find that pgr was reduced when population size increased (Table 3b, Figure S4). Thus, both study populations showed density‐dependent growth rates. However, we find no strong evidence of density dependence in the vital rates or in fecundity. In particular, the negative correlations suggesting density dependence in juvenile and adult apparent survival in the study population in Vaasa were of much lower magnitude or even reversed in sign in the study population in Luoto (Table 3b). The only consistent pattern across the two study populations was that the proportional contribution made by immigration to next year's population size (i.e., immigration rate) clearly declined with an increase in population size (Table 3b). However, the density dependence in immigration rate was not caused by less immigrants arriving in the population when population size was larger, as the number of immigrants did not correlate significantly with population size (Table 3, Figure S4). Instead, immigration rate declined in larger populations, because approximately the same number of immigrants contribute proportionally less in a larger than in a smaller population size (immigration rate was calculated as the number of migrants establishing at time *t *+* *1 per individual in the population at time *t*).

### 3.5 Relationship in demographic rates between study populations

The annual population growth rates in the two study populations did not correlate (Table 4, Figure S5a). Neither annual juvenile, nor adult apparent survival rates correlated between the study populations (Table 4, Figure S5b,c). Lastly, the immigration rates did not correlate between the two study populations (Table 4, Figure S5d). These findings indicated that the two study populations had uncoupled dynamics and demographic rates.

**Table 4:** The correlation and its 95% CRI between annual values for different parameters (par) estimated for the Vaasa and Luoto population for 2002–2013. The data are plotted in Figure 4

Parameters	Correlation	Lower 95% CRI	Upper 95% CRI	*p* (*r* > 0)
Pop growth rate	−.096	−0.51	0.34	.36
Juvenile survival	.023	−0.45	0.50	.53
Adult survival	.11	−0.39	0.57	.65
Immigration rate	−.015	−0.48	0.48	.46

## 4 Discussion

We constructed IPM based on long‐term data collected in two nest‐box study populations of individually marked flying squirrels. The IPM estimated all demographic rates underlying population dynamics in these study populations, including immigration. Our findings regarding capture and apparent survival probabilities are in agreement with earlier analysis (Lampila et al., 2009). We find that annual differences in fecundity and survival do not impact strongly on population growth rate. Instead, population growth rates are strongly associated with immigration rates in both populations. The population growth rate is furthermore subjected to density dependence. A striking feature of all demographic rates and of the population growth rate is that these do not correlate between the two study populations, despite their geographic proximity (100 km). Indeed, we find that one study population shows a stochastic decline, whereas the other one displays stochastic fluctuations around equilibrium. We therefore conclude that Siberian flying squirrel subpopulations have largely uncoupled dynamics, with each of the subpopulations critically depending on immigrants arriving from the other subpopulations.

Interannual variation in flying squirrel species abundance is commonly observed (Gomez et al., 2005; Fryxell et al., 1998; Lampila et al., 2009; Priol et al., 2014). We find clear evidence that immigration is the most important component regulating the population dynamics in flying squirrels. The estimation of immigration rate in the IPM is inferred from the difference in the observed population dynamics to the dynamics as they are estimated to be without immigration on the basis of data‐derived fecundity and survival estimates (Abadi et al., 2010). Because it is an inferred rate, it has relatively high uncertainty and hence wide confidence intervals. Despite this uncertainty, there is a strong association between immigration rate and population growth rate in studied populations. In fact, we find a strong regulatory role of immigrants for local population dynamics, which is consistent with IPM‐based studies conducted in some bird populations (Schaub et al., 2012, 2013), although not all studies find that immigration is important (e.g., Demerdzhiev, Stoychev, Dobrev, Spasov, & Oppel, 2015; Lieury, Gallardo, Ponchon, Besnard, & Millon, 2015). For small‐ or medium‐sized mammals, we are unaware of studies that use IPMs to link immigration to population dynamics. However, it is clear that dispersal potentially plays an important role in dynamics of mammalian populations (Bowers, 1997; Debeffe et al., 2012; Selonen & Hanski, 2004; Stenseth & Lidicker, 1992; Telfer, Holt, Donaldson, & Lambin, 2001).

A striking feature in our flying squirrel study populations is that the number of immigrants is approximately equal across population sizes. As a consequence, immigration plays a crucial role in returning population size to larger numbers after a reduction in population size. Apparently, the potential for a young female to establish as a breeder is largely unaffected by the number of adult females already present in the study population. This finding likely reflects the mating system of flying squirrels, where females occupy relatively small (7 ha) home ranges (Selonen et al., 2013). Because flying squirrels’ movement is not hampered by landscape structure (Selonen & Hanski, 2004, 2012), we believe the number of immigrants settling in the study population is largely determined by the population sizes in the surrounding contiguous area. Our estimates of survival and fecundity clearly demonstrate that without immigration, the local recruitment of offspring would be insufficient to replace the annual loss of adult females due to mortality. Importantly, these findings do not imply that we are dealing with sink populations; instead, they reflect the fact that on the level of the landscape, there exists a network of subpopulations (Runge, Runge, & Nichols, 2006; Thomas & Kunin, 1999) which are connected by movement of flying squirrel individuals, which radiotracking studies have demonstrated to be mainly juveniles (Hanski & Selonen, 2009).

Our study populations are two networks of nest boxes. Thus, they cover only part of the landscape, and flying squirrels nesting outside a nest‐box network are not part of the study population, even if they nest at close proximity to the nest‐box network. Thus, a female immigrating into the study population need not have come from a large distance. Nevertheless, natural cavities are not common in the commercially managed forests of Finland (<0.1 natural cavity per hectare, own observation), and adult flying squirrel females show high site tenacity (Selonen et al., 2013). It should also be noted that in our study even at high flying squirrel population size, large majority of boxes (70%–80% of all boxes) were empty. Thus, number of available nest boxes was not limiting breeding or immigrating individuals. All else being equal, a reduction in the spatial scale of a study area will increase the strength of immigration affecting the population (Lambrechts, Visser, & Verboven, 2000; Thomas & Kunin, 1999). We note, however, that the spatial extent of both our study populations is reasonably large (25–44 km^2^), and thus, our nest‐box networks cover a large amount of potential home ranges and distances typically dispersed by female flying squirrels (around 2 km; Hanski & Selonen, 2009). Thus, we view the importance of immigration for local population dynamics as part of the population ecology of flying squirrels, and not merely as an outcome of the scale or design of this study.

While these above considerations underline the intuitive importance of immigration in this system, it is perhaps less clear why other demographic rates show no or only a weak association with population growth rate. One possibility is that our surveys underestimate the reproductive output of females, especially that of second broods (see 2). On the other hand, fecundity is the demographic rate that shows a very low correlation to population growth rate in both populations (*r* ≤ .1), and—although we cannot formally exclude the possibility—we believe it is unlikely that putative census‐related biases would qualitatively change our conclusion regarding the importance of fecundity in regulating flying squirrel population dynamics. Earlier studies have shown that breeding dispersal in flying squirrels, at least in females, is practically absent (Hanski & Selonen, 2009; Selonen, & Wistbacka, 2017). From this perspective, the lower apparent survival of juveniles compared with adult flying squirrels is partly due to a higher probability to emigrate out of the study population for juveniles compared with adults. A problematic issue is that our estimates of apparent survival have ignored the loss of tags which we know happens in approximately 3% of recaptures. As a consequence, our apparent survival estimates are conservative and the estimated number of immigrants may be upward‐biased. Tag loss can be incorporated into capture–recapture models of apparent survival, but this requires individuals to have been marked by a second independently operating technique (Pollock et al., 1990). Such a study was here not conducted, but appears highly worthwhile. Nevertheless, this relative modest rate of tag loss presents a source of error noise, rather than a source of bias, from the perspective of exploring the importance of the demographic rates for population growth. This is because there is no reason to expect that the annual probability for tag loss (and the bias this potentially creates in estimates of demographic rates) covaries with changes in population size. We expect that majority of tag loss resulted from juveniles losing the tag within natal nest. Flying squirrels do not, for example, perform behaviors like fighting where they might lose the tag.

### 4.1 Population dynamics

The population growth rates and the demographic rates underlying it did not correlate in our two study populations. Consequently, these rates are unlikely to be determined by factors which are spatially correlated over the distance of about 100 km which separated the two study populations. In general, one would expect synchrony in dynamics of spatially separated populations to be forced over some distances by, for example, climatic conditions (Moran, 1950) or dispersal (Ranta et al., 1995). This finding suggests that the demographic rates in our two flying squirrel study populations are strongly affected by local conditions. Examples of local factors could be the annual density of flying squirrel predators (e.g., avian predators) in the study population, local alterations in the habitat (e.g., by forestry, Santangeli, Wistbacka, Hanski, & Laaksonen, 2013), or availability of local food resources (Selonen, Wistbacka, & Korpimäki, 2016). At the same time, each study population is clearly dependent on the immigration of individuals from elsewhere. Our finding of uncoupled dynamics is tentative, as it is based on only two populations, and hence needs to be confirmed by a comparison of multiple populations. Nevertheless, its implications for the conservation of the species are important. Firstly, assessing the conservation status of a species with uncoupled local dynamics requires a larger, and hence, more expensive, monitoring scheme compared to a situation where spatial synchrony is sufficiently high to be certain that the dynamics of a local population is representative of the surrounding populations. Second, a lack of correlation in population dynamics over larger distances suggests it is hard to generalize findings made in one population. From this perspective, our finding that demographic and population growth rates in two populations do not covary underlines the importance of studying more than one population in order to obtain population ecological insights.

## 5 Conclusion

We used exceptionally extensive long‐term mark–recapture data set to study relative roles of different demographic rates behind population growth in a forest‐dwelling rodent. Our study highlights the role of knowledge on dispersal, for example, for effective conservation management (Driscoll et al., 2014). We conclude that immigration may be the driving force of population growth also in species other than those that can fast move over large areas, such as birds (Schaub et al., 2012, 2013).

## Conflict of Interest

None declared.
