# Supplementary material for: Immigration ensures population survival in the Siberian flying squirrel
Source: Ecol Evol. 2017 Feb 15;7(6):1858–68. doi: 10.1002/ece3.2807 (PMC5355189; doi:10.1002/ece3.2807)
Supplement: Supplementary file 1 [file ECE3-7-1858-s001.docx]

ELECTRONIC SUPPLEMENT

belonging to the article “Efficient dispersal ensures population survival in the Siberian flying squirrel” by JE Brommer, R Wistbacka and V Selonen, containing the following material:

Fig S1. Annual fecundity (daughters per female) with its 95% CRI, as well as overall fecundity (dashed line) with its 95% CRI (see Table 1 in main text) for populations Vaasa and Luoto respectively.

Fig S2. Plots of the correlation between annual population growth rate and all demographic rates as derived from the Integrated Population Model for the population Vaasa. The statistics for these correlations are given in Table 3 in the main text.

Fig S3. Plots of the correlation between annual population growth rate and all demographic rates as derived from the Integrated Population Model for the population Luoto. The statistics for these correlations are given in Table 3 in the main text.

Fig. S4. Plots of the correlation between annual population growth rate (a,b) and immigration rate (c,d) and total population size as derived from the Integrated Population Model for the population Vaasa (a,c) and Luoto (b,d). The statistics for these correlations are given in Table 3 in the main text.

Fig. S5. Relationship between model derived estimates for the study populations in Vaasa and Luoto, for (a) annual population growth rate (pgr), (b) juvenile apparent survival, (c) adult apparent survival and (d) immigration rate. Data is plotted for each of the study years in common (2002 – 2013), with, as filled dots, the annual posterior modes. The 95% CRI are plotted as vertical and horizontal bars. See Table 4 for statistics on the correlation coefficients based on these model output.

Text S1. Details of the IPM

Text S2. IPM model in WinBUGS language

Fig S1.

Fig. S2.

Fig S3

Figure S4

Figure S5

**Text S1. Details of the IPM**

The IPM used in this paper is nearly identical to the one developed by Kéry& Schaub (2012), except two aspects. First, we did not assume that capture probability is constant, but allowed it to vary randomly across years. Second, we modeled immigration as the number of individuals originating from elsewhere entering the study population each year, and from that estimated annual immigration rate. Schaub & Fletcher (2015) demonstrated that this approach to model immigration was superior over modelling immigration as a rate directly (as in Kéry & Schaub 2012).

The IPM considered that changes in population size (number of females) arose because of four demographic processes: survival, fecundity, immigration and emigration. For each year *t*, we distinguished the number of locally recruited one-year old female flying squirrels at time *t* (*N*_1,t_) from the number of females older than 1 year (adults) that remained in the study area (*N_ad,t_*), and (one-year old or older) females which immigrated into the study area (*N_im,t_*). We assumed that the population size of adult females surviving and remaining in the study area (i.e. not emigrating) was defined by a binomial process

, (1)

where$\varphi_{ad,t}$ denotes the apparent survival of adult (≥ 1 year old) flying squirrel females from year *t* to *t*+1. All stages (one-year olds, adults, immigrants) were assumed to reproduce and have equal capacity to produce offspring, such that the number of one-years which recruited into the local population in the following year was defined as

$N_{1,t+1}\sim\mathrm{Poisson}\left(\left(N_{1,t}+N_{ad,t}+N_{im,t}\right)\varphi_{juv,t}f{}_{t}\right)$, (2)

where $\varphi_{juv,t}$denotes the apparent survival probability of a daughter to become a 1-year old in the study population at time *t*+1 and *f_t_* the number of juvenile daughters produced by a female at time *t* (fecundity). Note that apparent survival is the product of true survival and the probability to not emigrate out of the study area and emigration is hence implicitly included in this estimate.

Apparent survival $\varphi_{juv,t}$ and $\varphi_{ad,t}$ were estimated using a Cormack-Jolly-Seber (CJS) capture-recapture model. Following standard capture-recapture modeling procedure, information on a binary-coded encounter history of individuals for each calendar year was used to separate apparent survival from capture probability. Recapture probability was assumed to vary over the years.For estimating the survival rate of juveniles born at year *t* to become a one-year old at time *t*+1, $\varphi_{juv,t}$ , we considered the annual encounter history of all individuals tagged as a juvenile. These juveniles were either never encountered again after marking, or we included their encounter history up to and including their first recapture. In the CJS model for juvenile survival, the survival probability for a juvenile born at time *t* for the time interval *t* + *x* (*x* ≥ 1) to *t* + *x* +1 was assumed to be $\varphi_{ad,t}$. That is, after surviving its first winter, a juvenile has survival probability equal to adult apparent survival. The first and all subsequent recapture of tagged juveniles (which, by definition, all concern the individual as an adult), together with the encounter histories of females first tagged as an adult were considered as encounter histories in the estimation of $\varphi_{ad,t}$. Hence, all available encounter histories of tagged females were included. Between-year variation in survival was considered as a deviation from mean survival, such that for age-group *a* (*juv* or *ad*),

$\mathrm{logit}\left(\varphi_{a,t}\right)=\mathrm{logit}\left(\mu_{\varphi_{a}}\right)+\varepsilon_{\varphi_{a},t}$, (3)

where $\mu_{\varphi_{a}}$denotes the age-group specific mean survival and $\varepsilon_{\varphi_{a},t}$ the age-group and year specific deviation from $\mathrm{logit}\left(\mu_{\varphi_{a}}\right)$, which values were assumed to follow a normal distribution with zero mean and a standard deviation of σ_ɸa_. Likewise, annual difference in capture probability *p* at time *t* were assumed to follow from a normal distribution, such that

$\mathrm{logit}\left(p_{a,t}\right)=\mathrm{logit}\left(\mu_{p_{a}}\right)+\varepsilon_{p_{a},t}$, (4)

where$\mu_{p_{a}}$denotes the age specific mean capture probability and $\varepsilon_{p_{a},t}$ the age-group specific year-specific deviation from $\mathrm{logit}\left(\mu_{p_{a}}\right)$. The values of this deviation were assumed to follow a normal distribution with zero mean and standard deviation of $\sigma_{p_{a}}$.

The CJS model was fitted using a multinomial model based on the m-array of the encounter histories. For further details of this procedure, see Kéry & Schaub (2012).

Fecundity *f_t_* was considered a latent variable and was estimated by considering that

$J_{t}\sim\mathrm{Poisson}(R_{t}f_{t})$, (5)

where *J_t_* was the observed number of female juveniles which were tagged in year *t* and for who the identity of the mother was known and *R_t_* was the total number of females recorded to reproduce at time *t*, independently of how many marked daughters they produced. Given that *R_t_* was measured by the subset of females which were identified as mothers, the observed total production of juvenile daughters *J_t_* was considered a Poisson-distributed realization of the expected total female fecundity *R_t_ f_t_*. Thus, *f_t_* was assumed to be the same for detected and non-detected females present in the population at time *t* and of all age classes. Between-year variation in fecundity was modelled as

$\log(f_{t})=\log\left(\mu_{f}\right)+\varepsilon_{f,t}$, (6)

where$\log\left(\mu_{f}\right)$denotes the natural logarithm of average fecundity and $\varepsilon_{f,t}$ the year-specific deviation from this value. The latter was assumed to stem from a normal distribution with standard deviation σ_f_.

The total population size followed from the summation

$N_{tot,t}=N_{1,t}+N_{ad,t}+N_{im,t}$, (7)

where$N_{im,t}$ was the number of immigrant establishing themselves in year *t*. In order to make the immigration process comparable to survival and fecundity, the year-specific immigration was calculated as a derived parameter, by also expressing immigration as a rate, ι*_t_*. This immigration rate denoted the probability that a potential immigrant enters the population in the interval *t* to *t*+1 per adult female at time *t*. Thus,

$i_{t}=\frac{N_{im,t+1}}{N_{tot,t}}$. (8)

Defining immigration as the number of immigrants with a uniform prior that includes negative values, as we do here, has been shown to produce unbiased estimates of immigration (and therefore also of other demographic rates) in an IPM (Schaub & Fletcher 2015).

Equations (1), (2), and (7), and their hyperparameters as outlined above, define the system process in a state-space model of the dynamics of female flying squirrels. The state-space model was linked to census information assuming the observation process

$y{}_{t}\sim\mathrm{Poisson}(N_{tot,t})$, (10)

where *y_t_* denotes the census value of annual number of females. By specifying a Poisson observation process (eq10), we assumed that the relative observation error is constant (Poisson variance increases linearly with latent total population size), following Kéry & Schaub (2012) who noted that this formulation produced qualitative the same parameter estimates as the assumption of log-normal observation errors (as in Schaub et al. 2012). As census of the number of females per year, we tallied the number of observed nests with offspring per year, independently on whether the mother of the offspring was identified. Within the context of a state-space model, this is a reasonable census for the number of females since the observation of a nest with offspring implies a female is present. Furthermore, both positive deviations from the latent (true) number are possible (double counting; a female has two broods), but also negative deviations from the latent number are possible (female alive, but not breeding), which is a typical assumption in state-space models.

Annual growth rate λ_t_, was defined as

$\lambda_{t}=\frac{N_{tot,t+1}}{N_{tot,t}}$. (11)

In addition, the geometric mean growth rate over all *n* study years was calculated as$\mathrm{GM}\left(\lambda\right)=\exp\left(\frac{1}{(n-1)}\sum_{t=1}^{n-1} \ln\left(\lambda_{t}\right)\right)$. (12)

Immigration is an estimable quantity in the above outlined IPM, despite the fact that immigrants themselves are not identifiable (Abadi et al. 2010). Thus, no inferences are made by the researchers on which individuals in the data are immigrants. The number of immigrants is strictly inferred from the above listed equations. The model further does not assume that all juveniles are marked nor that all adults are captured each year. Demographic rates, including immigration, can be estimated because there is on the one hand information on the dynamics of the total population size (number of nests per year) and, on the other hand, detailed information on the demographic processes which relate to philopatric individuals (eq. (1) and (2)). The difference between these two components is here used to estimate immigration (see Abadi et al. (2010) for further details).

The values used for *y*, *R* and *J*, as defines above, are given in Table 1 in the main text.

A script of the here described integrated population model in the BUGS language is provided in Text S2.

**Text S2. IPM model in WinBUGSlanguage**

model {

#------------------------------------------------------------

# Integrated population model

# - Age structured model with 2 age classes:

# 1-year old and adult (at least 2 years old)

# - Age at first breeding = 1 year

# - Prebreeding census, female-based

# - All vital rates are assumed to be time-dependent (random)

# - Capture probability (p) assumed to be time-dependent (random effect)

# - Explicit estimation of immigration as number of immigrating females

#-------------------------------------------------------------

#----------------------------------------

# 1. Define the priors for the parameters

#----------------------------------------

# Initial population sizes

N1[1] ~ dnorm(10, 0.0001)I(0,) # 1-year old individuals

NadSurv[1] ~ dnorm(10, 0.0001)I(0,) # Adults >= 2 years

# Mean demographic parameters (on appropriate scale)

# Bounded to help with convergence

l.mphij ~ dnorm(0, 0.0001)I(-10,10)

l.mphia ~ dnorm(0, 0.0001)I(-10,10)

l.mfec ~ dnorm(0, 0.0001)I(-10,10)

l.p ~ dnorm(0, 0.0001)I(-10,10)

# Precision of standard deviations of temporal variability

sig.phij ~ dunif(0, 10)

tau.phij<- pow(sig.phij, -2)

sig.phia ~ dunif(0, 10)

tau.phia<- pow(sig.phia, -2)

sig.fec ~ dunif(0, 10)

tau.fec<- pow(sig.fec, -2)

sig.p ~ dunif(0, 10)

tau.p<- pow(sig.p, -2)

#immigration with uniform prior that includes zero immigrants

for (t in 1:nyears){

Nadimm[t] ~ dunif(-10, 30)

}#t

# Distribution of error terms (Bounded to help with convergence)

for (t in 1:(nyears-1)){

epsilon.phij[t] ~ dnorm(0, tau.phij)I(-15,15)

epsilon.phia[t] ~ dnorm(0, tau.phia)I(-15,15)

epsilon.fec[t] ~ dnorm(0, tau.fec)I(-15,15)

epsilon.p[t] ~ dnorm(0, tau.p)I(-15,15)

}

#-------------------------

# 2. Constrain parameters

#-------------------------

for (t in 1:(nyears-1)){

logit(phij[t]) <- l.mphij + epsilon.phij[t] # Juv. apparent survival

logit(phia[t]) <- l.mphia + epsilon.phia[t] # Adult apparent survival

log(f[t]) <- l.mfec + epsilon.fec[t] # Productivity

logit(p[t]) <- l.p + epsilon.p[t] # Recapture probability

} #t

#-----------------------

# 3. Derived parameters

#-----------------------

mphij<- exp(l.mphij)/(1+exp(l.mphij)) # Mean juv survival probability

mphia<- exp(l.mphia)/(1+exp(l.mphia)) # Mean adult survival probability

mfec<- exp(l.mfec) # Mean productivity

for (t in 2:nyears){

iota[t-1] <- Nadimm[t]/Ntot[t-1] #immigration rate as derived par

} #t

# Annual Population growth rate

for (t in 1:(nyears-1)){

lambda[t] <- Ntot[t+1] / Ntot[t]

logla[t] <- log(lambda[t])

}

# Geometric mean population growth rate over all years

mlam<- exp((1/(nyears-1))*sum(logla[1:(nyears-1)]))

#--------------------------------------------

# 4. The likelihoods of the single data sets

#--------------------------------------------

# 4.1. Likelihood for population population count data (state-space model)

# 4.1.1 System process

for (t in 2:nyears){

mean1[t] <- f[t-1] * phij[t-1] * Ntot[t-1]

N1[t] ~ dpois(mean1[t])

NadSurv[t] ~ dbin(phia[t-1], Ntot[t-1])

} #t

# 4.1.2 Observation process

for (t in 1:nyears){

Ntot[t] <- NadSurv[t] + Nadimm[t] + N1[t]

y[t] ~ dpois(Ntot[t])

} #t

# 4.2 Likelihood for capture-recapture data: CJS model (2 age classes)

# Multinomial likelihood

for (t in 1:(nyears-1)){

marray.j[t,1:nyears] ~ dmulti(pr.j[t,], r.j[t])

marray.a[t,1:nyears] ~ dmulti(pr.a[t,], r.a[t])

}#t

# Calculate number of released individuals

for (t in 1:(nyears-1)){

r.j[t] <- sum(marray.j[t,])

r.a[t] <- sum(marray.a[t,])

}#t

# m-array cell probabilities for juveniles

for (t in 1:(nyears-1)){

q[t] <- 1-p[t]

# Main diagonal

pr.j[t,t] <- phij[t]*p[t]

# Above main diagonal

for (j in (t+1):(nyears-1)){

pr.j[t,j] <- phij[t]*prod(phia[(t+1):j])*prod(q[t:(j-1)])*p[j]

} #j

# Below main diagonal

for (j in 1:(t-1)){

pr.j[t,j] <- 0

} #j

# Last column

pr.j[t,nyears] <- 1-sum(pr.j[t,1:(nyears-1)])

} #t

# m-array cell probabilities for adults

for (t in 1:(nyears-1)){

# Main diagonal

pr.a[t,t] <- phia[t]*p[t]

# above main diagonal

for (j in (t+1):(nyears-1)){

pr.a[t,j] <- prod(phia[t:j])*prod(q[t:(j-1)])*p[j]

} #j

# Below main diagonal

for (j in 1:(t-1)){

pr.a[t,j] <- 0

} #j

# Last column

pr.a[t,nyears] <- 1-sum(pr.a[t,1:(nyears-1)])

} #t

# 4.3. Likelihood for productivity data: Poisson

for (t in 1:(nyears-1)){

J[t] ~ dpois(rho[t])

rho[t] <- R[t] * f[t]

} #t

} #end model
